# Supplementary material for: Interplay of the two ancient metabolites auxin and MEcPP regulates adaptive growth
Source: Nat Commun. 2018 Jun 11;9:2262. doi: 10.1038/s41467-018-04708-5 (PMC5995930; doi:10.1038/s41467-018-04708-5)
Supplement: Supplementary file 1 — Supplementary Information [file 41467_2018_4708_MOESM1_ESM.pdf]

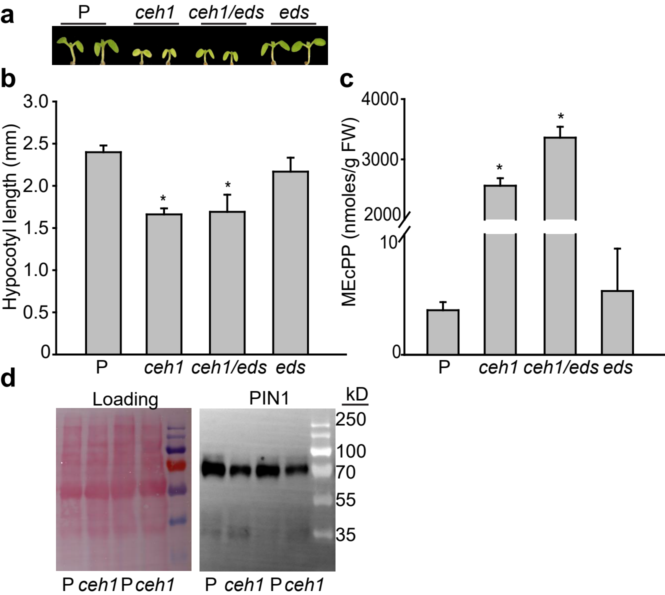

Supplemental Figure 1. *Ceh1* retarded growth is salicylic acid independent.

(**a**) Representative images of P, *ceh1*, *ceh1/eds16*, and *eds16* seedling depicting hypocotyl phenotypes, and (**b**) the respective quantifications measured in n≥45, performed on 7-day old seedlings grown under 16 h light / 8 h dark, and at ~20-22 °C. (**c**) MEcPP levels in aforementioned genotypes. (**d**) Uncropped western blot showing the Ponceau S stained membrane and PIN1 Western blot with molecular weight markers. Data are expressed as mean of six biological replicates ± SD. Asterisks denote significant differences from P as determined by Student's t tests ($P < 0.05$).

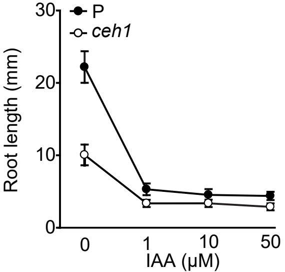

Supplementary Figure 2. IAA differentially inhibits root growth in *ceh1* and P

Quantitative measurements of root length in control (P) and *ceh1* seedlings grown in different IAA concentrations.

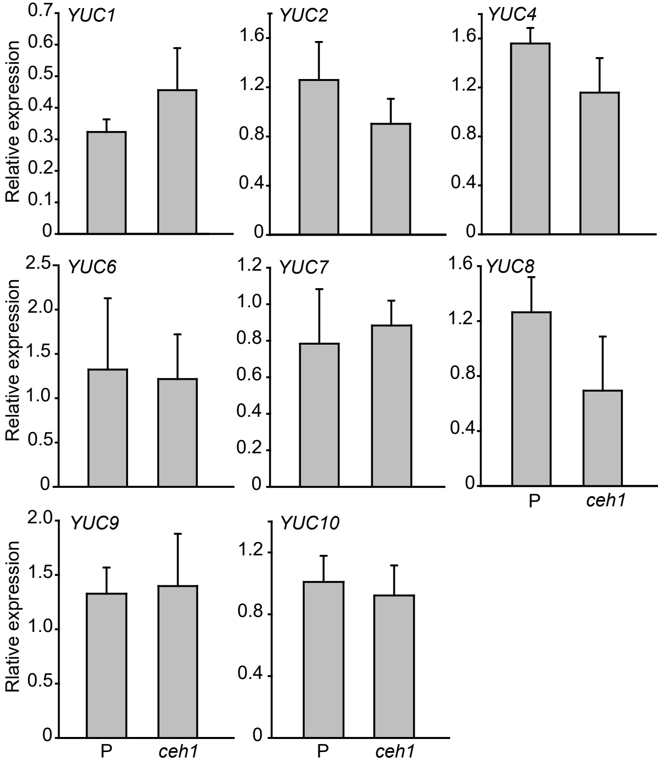

Supplementary Figure 3. Relative expression levels of *YUCs* in P and *ceh1* Total RNA extracted from P and *ceh1* seedlings were subjected to RT-q-PCR analysis. The mRNA levels of each of the *YUCs* genes were normalized to the levels of At4g34270 (T1P41-like family protein) and At4g26410 (M3E9). Data are mean fold difference ± SD of three biological replicates each with three technical repeats. Two-tailed Student's *t* tests showed no significant differences in the expression levels of these *YUCs* in P versus *ceh1* plants.

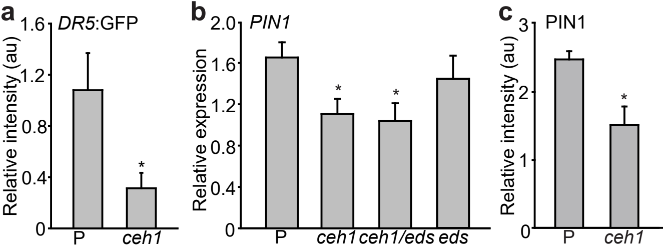

Supplementary Figure 4. *DR5*:GFP and PIN1 abundance are reduced in *ceh1*

(**a**) Quantification of *DR5* signal intensity in P and *ceh1* seedlings shown in Fig. 1**e**. (**b**) *PIN1* mRNA levels in P and *ceh1*, *ceh1/eds16* and *eds16* seedlings. Total RNA from each genotype was subjected to RT-q-PCR analysis. *PIN1* mRNA levels were normalized against mRNA levels of At4g34270 (T1P41-like family protein) and At4g26410 (M3E9) measured in the same samples. Data are expressed as mean fold difference ± SD of three biological replicates and three technical repeats each. Two-tailed Student's *t* tests showed no significant differences in the mRNA levels of *PIN1* in these genotypes. (**c**) Quantification of PIN1 Western blot signal intensity in P and *ceh1*.

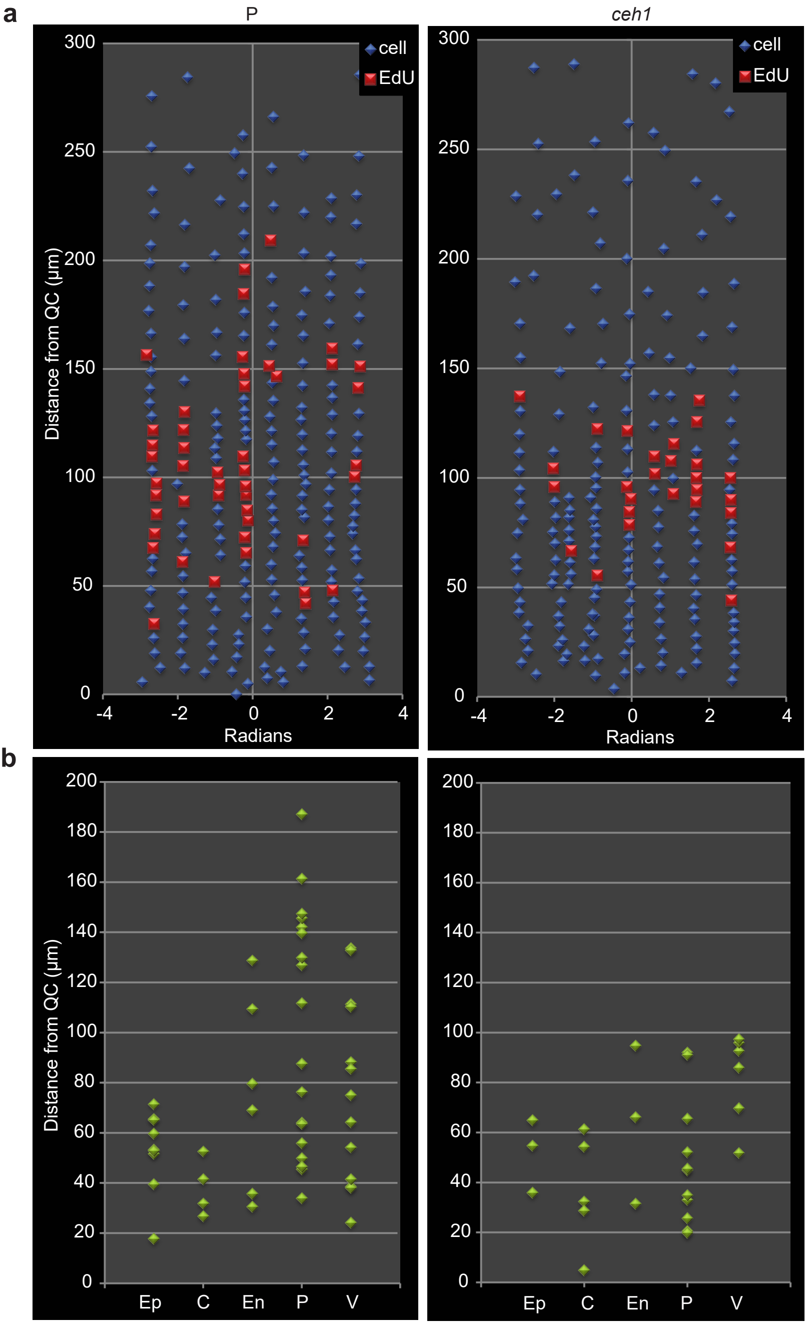

Supplementary Figure 5. The length of the replication zone and proliferation zone were reduced in *ceh1* mutant

DNA synthesis in proliferating root cells was monitored by EdU incorporation and subsequent confocal laser scanning microscopy. (**a**) Representative DNA replication maps of virtually 'un-rolled' cortex. X- axis- radians; Y- axis- distance from QC. Microscopically detected cells are labelled in blue. Proliferating cells were detected by EdU labeling and are shown in red. Unrolled cortex from typical roots is shown. (**b**) Map of the proliferation zone in P and *ceh1*. Green rhombs show distribution of mitosis events in representative root. Ep - epidermis; C - cortex; En - endodermis; P - pericycle; V - vascular tissue. Mitosis events were analysed in iRoCS ToolBox.

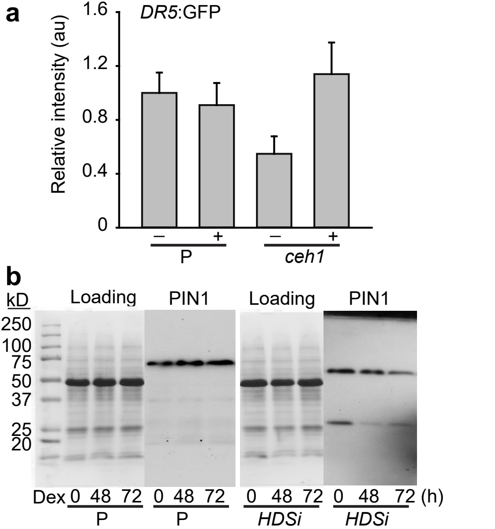

Supplemental Figure 6. Fosmidomycin (FSM) recovers *DR5*:GFP signals in *ceh1*.

(**a**) Quantification of DR5 signal intensity in P and *ceh1* seedlings shown in Fig. 2c. (**b**) Uncropped original Western blot showing the Ponceau S stained membrane and PIN1 Western blot with molecular weight markers.

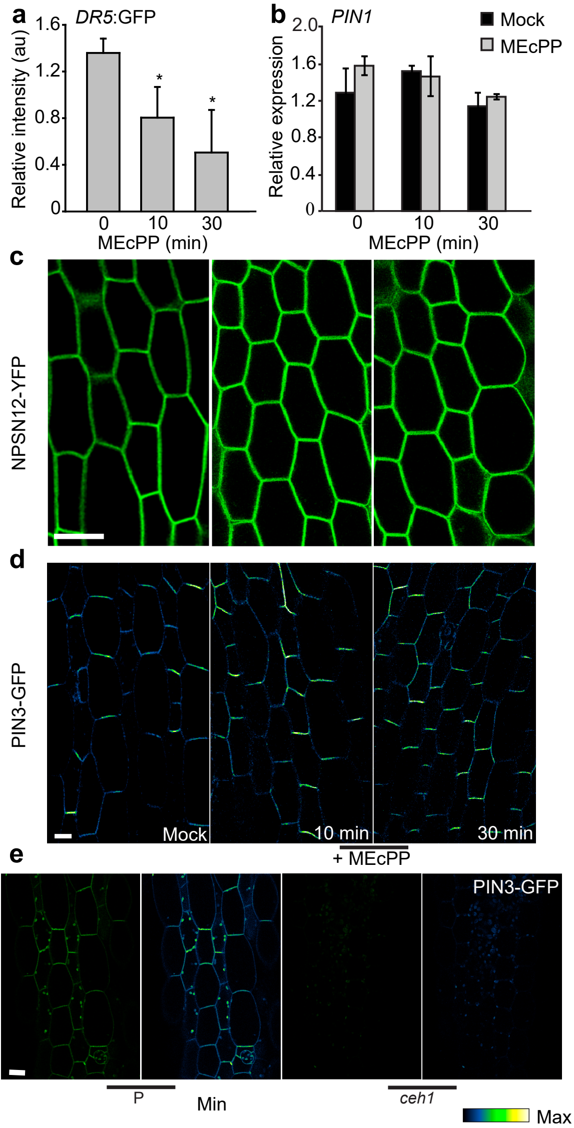

Supplementary Figure 7. MEcPP does not alter NPSN12 and PIN3 abundance

(**a**) Quantification of *DR5*:GFP after MEcPP application showed reduced signal intensity. Data are expressed as means ± SD of three biological replicates, each with 10 technical replicates. Asterisk indicates significant difference as determined by a two-tailed Student's *t* tests with a significance of P<0.05. (**b**) *PIN1* mRNA levels are not altered in response to exogenously applied MEcPP. Total RNA extracted from P seedling before and at intervals after MEcPP treatments were subjected to RT-q-PCR analysis. The mRNA levels were normalized against At4g34270 (T1P41-like family protein) and At4g26410 (M3E9) measured in the same samples. Data are expressed as mean fold difference ± SD of three biological replicates each with three technical repeats. Two-tailed Student's *t* tests showed no significant differences in the mRNA levels of *PIN1* in response to MEcPP treatment. (**c**-**d**) Representative images depicting fluorescence signal intensities of two plasma membrane proteins, NPSN12-YFP (**c**) and PIN3-GFP (**d**), in mock and MEcPP treated hypocotyls. Images are representative of three independent experiments, each with 10 biological replicates. (**e**) Representative images depicting reduced PIN3-GFP fluorescence signal intensity in hypocotyls of *ceh1* mutant as compared to P. Images are representative of two independent experiments, each with 10 biological replicates. The color-coded bar displays the PIN3 fluorescence.

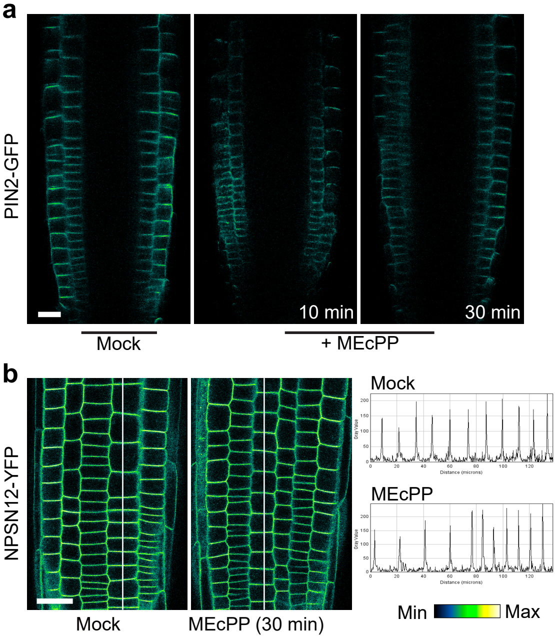

Supplementary Figure 8. MEcPP reduces PIN2 abundance slightly but does not alter NPSN12 in roots.
Representative images depicting fluorescence signal intensities of two plasma membrane proteins, (**a**) PIN2-GFP and NPSN12-YFP (**b**) in mock and MEcPP treated roots. Images are representative of at least 10 biological replicates. The color-coded bar displays PIN2 and NPSN12 fluorescence intensity. Scale bars: 20 μm

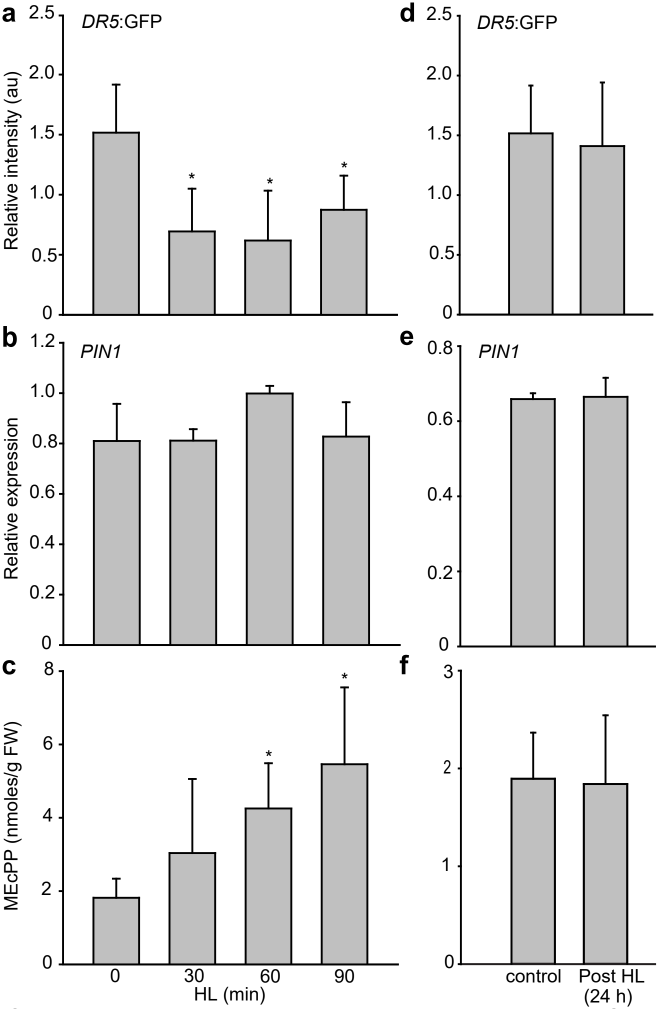

Supplemental Figure 9. High light treatment alters MEcPP and *DR5*:GFP levels.

Seedlings response at various time points after HL treatments (0, 30, 60, and 90 min and 24 h post treatment) as depicted by *DR5*:GFP signal intensities measured in two independent experiments in at least 15 biological replicates (**a**, **d**), *PIN1* mRNA levels were examined in three biological replicates, each with three technical replicates (**b&e**), and MEcPP levels were measured in two biological replicates, each with six technical replicates (**c&f**). Data are expressed as mean ± SD. Asterisk indicates significant difference P<0.05 determined by a two-tailed Student's *t* tests.

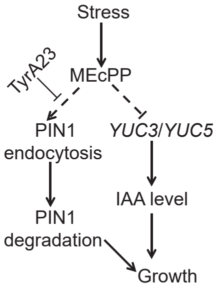

Supplemental Figure 10. Schematic model depicting MEcPP mode of action in regulating adaptive growth by dual transcriptional and post-translational regulatory inputs that modulate auxin and PIN1 abundance.
